# Temporal and spatial co-occurrence of pacific oyster mortality and increased planktonic *Vibrio* abundance

**DOI:** 10.1016/j.isci.2024.111674

**Published:** 2024-12-21

**Authors:** Elliot Scanes, Nachshon Siboni, Jaimie Potts, Shivanesh Rao, Maurizio Labbate, Justin R. Seymour

**Affiliations:** 1 Climate Change Cluster, University of Technology Sydney, Ultimo, NSW, Australia; 2 NSW Department of Climate Change, Energy, the Environment and Water, Parramatta, NSW, Australia; 3 School of Life Sciences, University of Technology Sydney, Ultimo, NSW, Australia

**Keywords:** Global change, Oceanography, Zoology, Aquatic biology

## Abstract

Oyster mortality and human food poisoning events are linked to pathogens from the *Vibrio* genus. However, the link between these events, planktonic bacterial dynamics and environmental variables has not yet been resolved. In Port Stephens, Australia, we characterized the microbial community and quantified the abundance of total *Vibrio*, *Vibrio harveyi,* and *Vibrio parahaemolyticus* in a (i) 27-month seawater planktonic microbial time-series; (ii) samples of Pacific oysters (*Crassostrea gigas*) during a mortality event and (iii) seawater samples following the mortality event. *Vibrio harveyi* and *V. parahaemolyticus* exhibited seasonal abundance, peaking during the summer months. Total *Vibrio* and *V. harveyi* in seawater were significantly greater at sites with high levels of oyster mortality and decreased 5-fold in the weeks following oyster mortality. Our findings provide evidence for the role of *Vibrio* in oyster mortality events and indicate that ocean warming and elevated phytoplankton may stimulate putative pathogens in the *Vibrio* genus.

## Highlights

• *Vibrio* bacteria can kill oysters on oyster farms and cause food poisoning
• Planktonic *V. harveyi* and *V. parahaemolyticus* exhibited seasonal abundance
• Planktonic *V. harveyi* was elevated during widespread oyster mortality
• We demonstrate the link between environment, planktonic *Vibrio* and oyster mortality

## Graphical abstract

**Figure:**
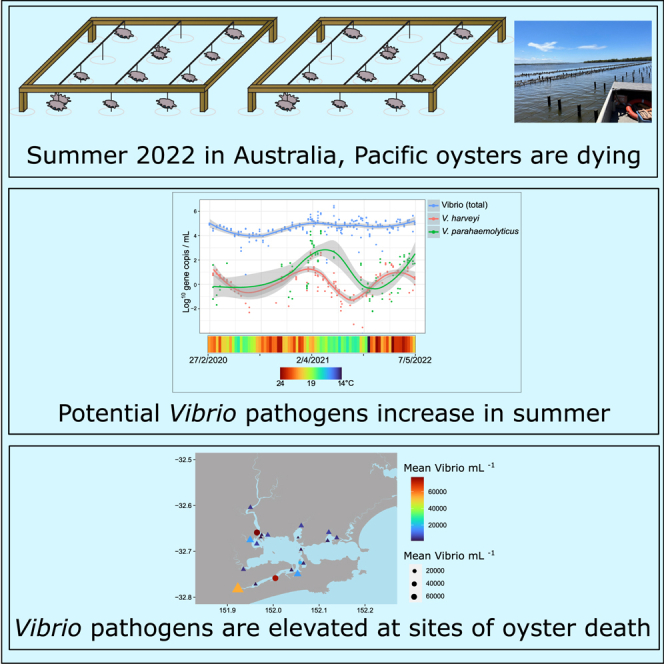

## Introduction

Oysters from the *Crassostrea* genus comprise 30% of all farmed molluscs, with 6 million tonnes of live oysters from this genus grown globally each year.^1^ The Pacific Oyster, *Crassostrea gigas*, is among the world’s most widely farmed seafoods.^1^ Pacific oysters are a vital link in the global food security chain, but their culture is significantly affected by disease outbreaks and mass mortality especially during warm weather.^2^^,^^3^ These mortality events have increased in frequency over recent decades to the point where the oyster industry is in a state of decline in many regions around the globe.^4^

Significant Pacific oyster mortality events have been reported globally. A recent major cause of these mortality events has been Pacific Oyster Mortality Syndrome (POMS),^3^ which is triggered following infection by Ostreid herpesvirus-1 (OSHV-1).^5^ The POMS virus first appeared causing significant mortality in France, before significant mortality occurring in other global locations in the proceeding years.^6^ While triggered by the OSHV-1 virus, there is evidence that POMS is polymicrobial disease, which also involves bacteria, in particular *Vibrio* spp. *Vibrio* are believed to play a role in oyster death,^3^^,^^5^ whereby the OSHV-1 virus impacts the oyster immune system, permitting infection by pathogenic *Vibrio*.^3^^,^^5^

Many Pacific oyster mortality events also transpire in the absence of OSHV-1, with these often occurring during periods of warmer temperatures, resulting in events that have become loosely termed “summer mortality” events.^7^^,^^8^^,^^9^^,^^10^^,^^11^ Summer mortality is a globally occurring disease with multiple lines of evidence suggesting that bacteria from the *Vibrio* genus are a likely causative agent.^7^^,^^8^^,^^12^

*Vibrio* pathogens pose a significant threat to both intensive aquaculture such as fish and prawn/shrimp farming,^13^^,^^14^ and less-intensive forms of aquaculture including bivalve farming.^5^^,^^15^^,^^16^ *Vibrio* are generally considered opportunistic pathogens of bivalves, proliferating and causing disease when the host’s immune system is compromised.^3^^,^^5^^,^^17^^,^^18^ Coastal warming triggered by climate change^19^^,^^20^ is suspected to be elevating the risk of *Vibrio*-associated disease and may be the cause of these increases in oyster mortality.^18^^,^^21^^,^^22^ Indeed, there is evidence that pathogenic *Vibrio*, including species from the *Vibrio harveyi* clade, may be responsible for oyster death following exposure to heat stress.^15^^,^^17^ *V. harveyi* can exert toxicity on oysters by producing serine proteases, metalloproteases, and cysteine proteases,^23^ with its pathogenicity enhanced at higher temperatures.^17^^,^^18^^,^^22^

There is also significant concern that warming oceans may be triggering increases in *Vibrio* that are pathogenic to humans.^24^ The pathogen *Vibrio parahaemolyticus* is the leading bacterial cause of seafood-borne disease globally, and can cause infections in humans when seafood, usually oysters, are consumed without adequate cooking.^25^^,^^26^ Globally, cases of *V. parahaemolyticus* infection have increased in recent decades,^27^ with elevated temperatures the most widely suspected cause.^28^

Given their significant impacts on both the health of aquaculture species, and the humans that consume them, understanding the environmental triggers for outbreaks of pathogenic vibrio has become an important goal within the aquaculture industry.^29^ The abundance of planktonic pathogens including *Vibrio* can be highly heterogeneous, whereby bloom events can occur due to favourable biotic and abiotic conditions including phytoplankton blooms and elevated temperatures.^30^^,^^31^^,^^32^^,^^33^ Importantly, however, it is unclear whether water-column blooms of putative pathogens translate into increased levels of oyster mortality or human health risks.

The most productive *C. gigas* growing region in mainland Australia is Port Stephens, with this region responsible for approximately 20% of all the state of New South Wales’s (NSW) oysters.^34^ However, this estuary has been impacted by significant oyster mortality events, including a notable event in the summer of 2013–2014, when there was up to 98% mortality at some locations.^7^ Subsequent microbiological examination of oysters from this event revealed *Vibrio*, specifically, *V. harveyi,* as a putative cause of oyster mortality.^7^ Another mass mortality of *C. gigas* occurred in Port Stephens in the summer of 2022–2023, when farmers reported mortality of up to 70%, with an estimated economic loss in the millions of Australian dollars, yet the causes of the event are currently unresolved.

To examine the potential causes of this period of elevated oyster mortality, we combined analysis of data from a long-term water sampling conducted in Port Stephens in the years preceding and during the mortality event, on-farm sampling during the mortality event, and subsequent sampling post-mortality event. We also examined the presence of the human pathogen *V. parahaemolyticus*. We hypothesized that elevated *Vibrio* and putatively pathogenic *V. harveyi* abundances in seawater would correlate with both periods of higher water temperatures and oyster mortality.

## Results

### Seawater timeseries shows seasonal *Vibrio* abundance

**Figure 1:**
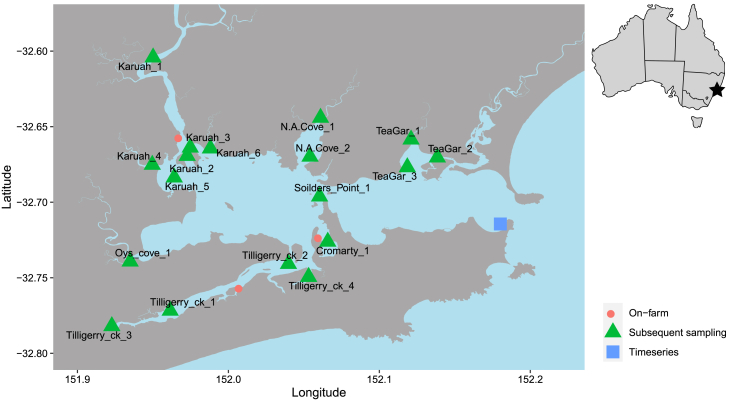
Sampling locations for each of the three sampling classes Tilligerry Creek and Karuah experienced high levels of mortality, while Cromarty Bay experience low levels. Small map of Australia indicates the position of Port Stephens on the coastline of New South Wales, Australia.

**Figure 2:**
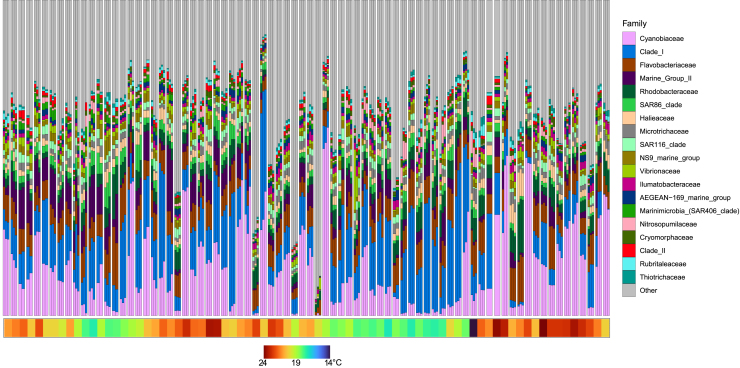
Barplot of the relative abundance of the 20 most abundant bacterial families from weekly seawater samples collected as part of the 27-month timeseries Gray sections are “other” taxa. Each bar is a replicate sample and the clusters are samples from those weeks. Bars are ordered by collection date. Color chart below indicates mean SST at the time of sampling.

**Figure 3:**
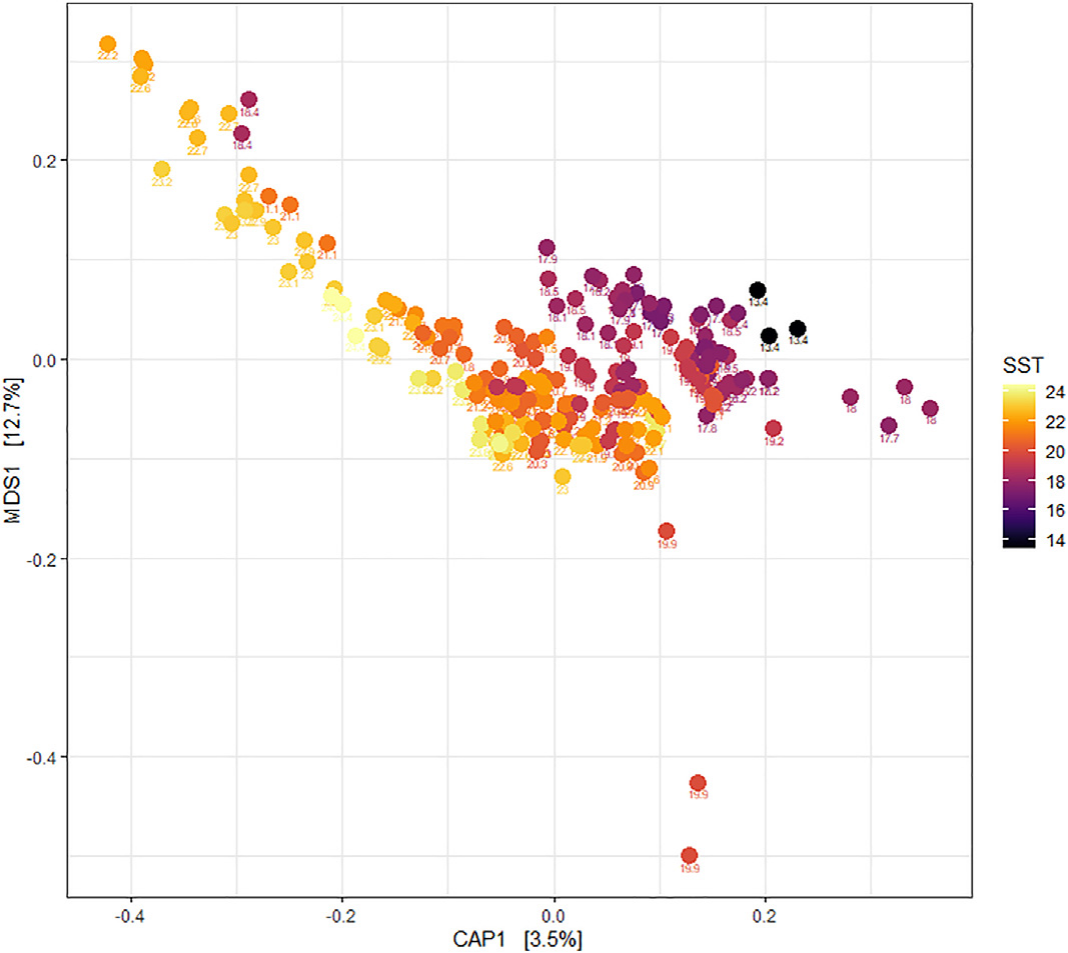
CAP ordination plot of bacterial communities the 27-month long seawater time series collected in Port Stephens NSW, color coded by SST

During the two-year seawater timeseries (Figure 1), the composition of the bacterial community changed significantly over time (PERMANOVA F_77,221_ = 2.7, *p* < 0.001). Changes in sea surface temperature (SST) were correlated with changes in seawater bacterial community composition (wunifrac distance matrix) (PERMANOVA F_1,221_ = 8.5, *p* < 0.001; Figures 2 and 3). We identified changes in abundance of 3,156 bacterial ASVs that were significantly correlated with changes in SST, including 69 *Vibrio* ASVs (DESEQ padj <0.05). Approximately half of these ASVs increased in abundance (1,624 ASVs), while half decreased in abundance (1532 ASVs) as SST increased.

We also found that the abundance of 643 bacterial ASVs were correlated with changes in the abundances of Chloroplast, including 19 *Vibrio* species (PERMANOVA F_1,221_ = 1.4, *p* < 0.005; DESEQ analysis *P*_adj_ < 0.05). Notably, this relationship was not caused by a simple decrease in relative abundance related to increased chloroplast sequences, because 98.9% of these bacterial ASVs increased in relative abundance as chloroplast counts increased.

**Figure 4:**
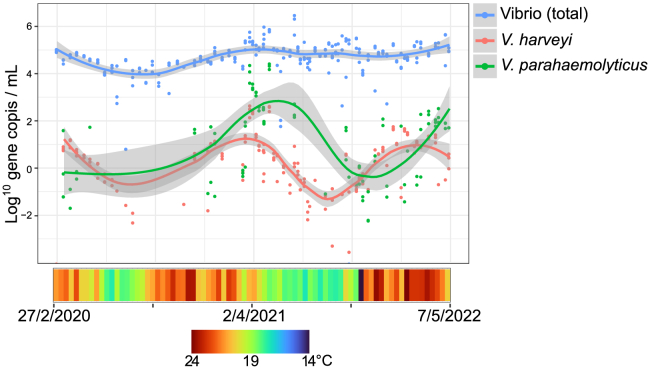
Log transformed abundance of total *Vibrio* (blue), *V. harveyi* (red) and *V. parahaemolyticus* (green) over 27 months of seawater monitoring in Port Stephens NSW Color chart below indicated SST at the time of sampling. Trend lines are represented by a loess smoothing function and the gray shaded areas indicate 95% confidence intervals.

During the 27-month times series, *Vibrio, V. harveyi* and *V. parahaemolyticus* abundances followed a cyclical pattern (GAM; “Day” *p* < 0.005; “Year” *p* > 0.05). Our GAM model showed that bacterial abundances significantly increased and decreased throughout the year in a pattern that was repeated across each of the years (2020, 2021 and 2022) (Figure 4). The greatest *V. harveyi* levels (355 ± 50 copies mL^−1^) were recorded in the months of March and April (Figure 4). In contrast, during August, September, October *V. harveyi* levels were below the detection limit. Similar patterns were also observed for *V. parahaemolyticus*, although, *V. parahaemolyticus* bacterial abundances peaked a few weeks after *V. harveyi.* These patterns were confirmed for both *V. harveyi* and *V. parahaemolyticus* by Random Forest models that revealed SST (*V. harveyi* MSE 11%; *V. parahaemolyticus* 11.8%), Chloroplast reads (*V. harveyi* MSE 15.8%; *V. parahaemolyticus* 13.7%) and Chlorophyll *a* (*V. harveyi* MSE 11%; *V. parahaemolyticus* 11.8%) were all significant predictors of bacterial abundance in the model.

Temporal patterns in total *Vibrio* were not as clearly defined as *V. harveyi* and *V. parahaemolyticus,* but cyclical patterns were still apparent (GAM; “Day” *p* < 0.005; “Year” *p* > 0.05; Figure 4). *Vibrio* concentrations ranged from below the detection limit in some months, to 1.8 × 10^6^ ± 7.5 × 10^5^ copies mL^−1^, in June 2021. Random Forest models revealed SST as the most important predictor (MSE 7%) of total *Vibrio* abundance, followed by Chlorophyll (MSE 6%).

### On-farm sampling (February) shows elevated *Vibrio* abundance

During February 2023 there was widespread occurrence of significant Pacific oyster mortality in Port Stephens. Oyster farmers in this region estimated mean mortality rates of 5% at Cromarty Bay, 70% at Tilligerry Creek and 75% at the Karuah River (Figure 1). Pacific oysters from Port Stephens are frequently monitored for OsHV-1 as part of a monitoring program, and as of the time of the study, OsHV-1 has never been detected.^35^

**Figure 5:**
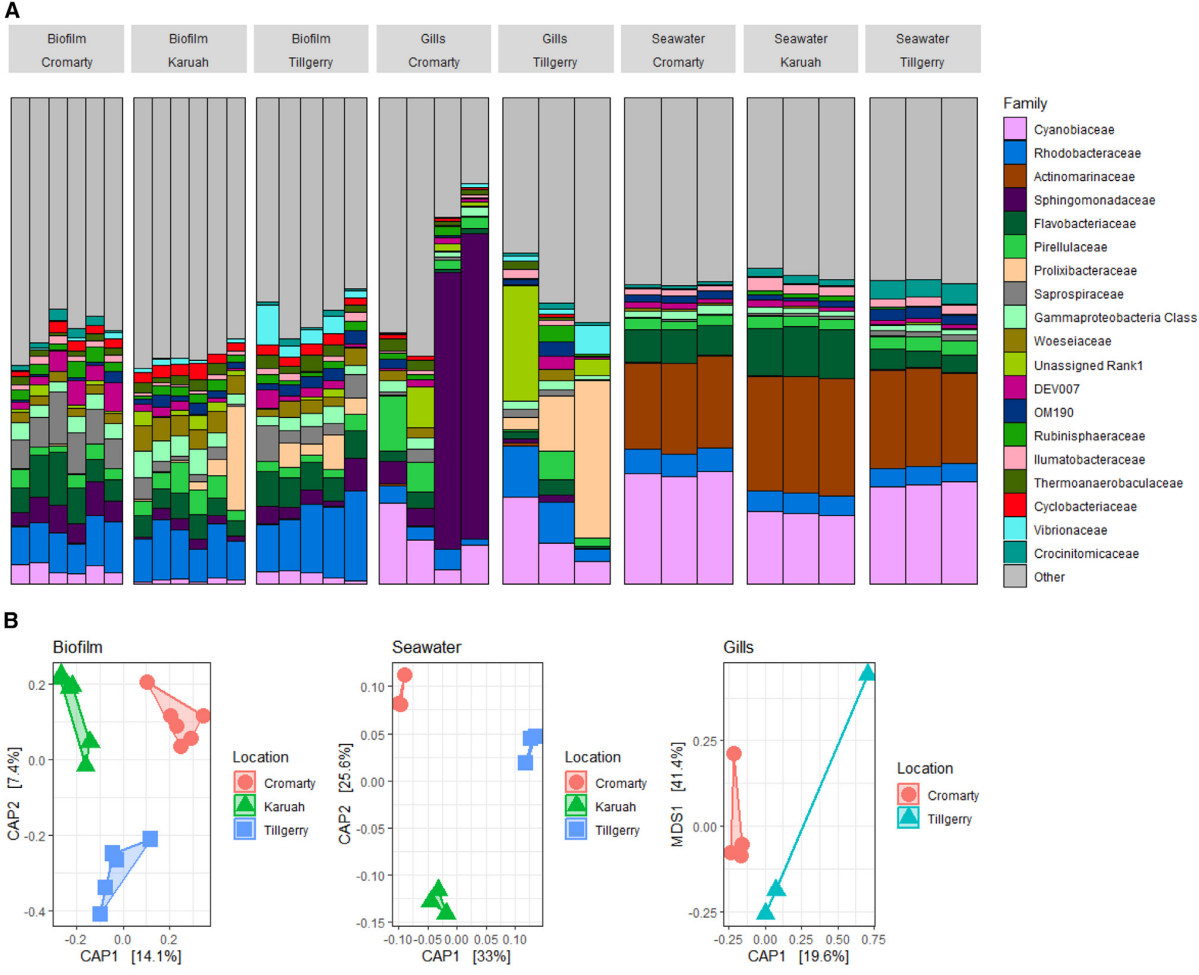
Bacterial communities from on-farm sampling (A) Barplot of the top 20 most abundant bacterial families from oyster biofilms, Gills and seawater from the sampling sites Cromarty Bay, Tilligerry Creek and Karuah River. Each bar represents an individual sample. (B) CAP ordination plots calculated from Weighted Unifrac distances of bacterial communities in each sample type (biofilm, gills, seawater). Locations are indicated by shapes and colors.

**Figure 6:**
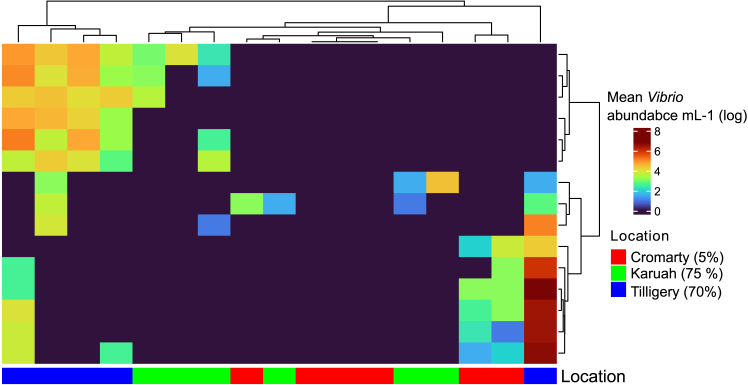
Heatmap of abundance of the 15 most abundant *Vibrio* ASVs in oyster biofilms (rows), grouped by taxonomic similarity Each column represents a replicate sample, with the location (and % oyster mortality at each location) indicated by color. Locations are grouped by similarity based on the *Vibrio* community. Similarity among rows and columns were calculated using hierarchical clustering with optimal leaf ordering, using Euclidean distances.

Each location and sample type (seawater, tissue, biofilm) harbored a unique microbial community (Figure 5). Microbial community composition significantly differed among locations for seawater samples (PERMANOVA, F_2,8_ = 4.8 *p* < 0.05) and biofilms (PERMANOVA, F_2,16_ = 1.9 *p* < 0.01). Among these seawater samples, there were 97 ASVs (DESEQ padj <0.05) that differed significantly in relative abundance between Cromarty Bay (low mortality) and Tilligerry Creek (high mortality) sites. The majority of these ASVs occurred in lower abundance at Tilligerry creek (66 ASVs). There were also 65 ASVs that differed significantly in relative abundance between Cromarty Bay (low mortality) and Karuah River (high mortality). Notably, the 15 most abundant *Vibrio* ASVs in oyster shell biofilms occurred in significantly higher abundance at the high mortality site at Tilligerry Creek compared to Karuah or Cromarty (Kruskal – Wallis; χ^2^ = 3729, *p* < 0.01; Figure 6). Among the oyster gill samples there were no significant differences in the composition of bacterial communities between locations (PERMANOVA, *p* > 0.05).

**Figure 7:**
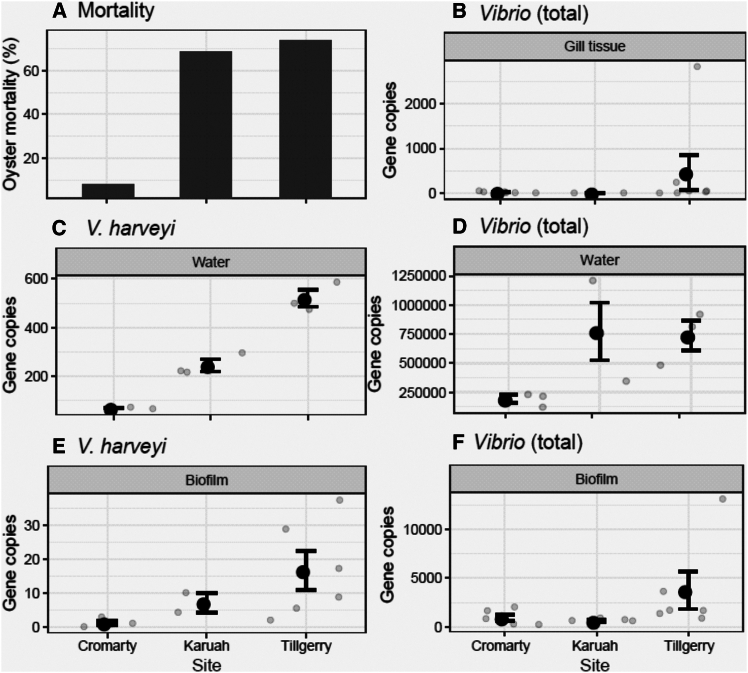
Bacterial abundances from on-farm sampling (A) Oyster mortality recorded by oyster farmers at the three sampling sites. (B) *Vibrio* (total) abundance (mg^−1^) in oyster gill tissue collected from the three sites. (C) *V. harveyi* abundance (L^−1^) in water samples collected from the three sites. (D) *Vibrio* (total) abundance (L^−1^) in water samples collected from the three sites. (E) *V. harveyi* abundance in oyster biofilm samples collected from the three sites and. (F) *Vibrio* (total) abundance in oyster biofilm samples collected from the three sites. All samples were collected on-farm in March 2023. Small gray points on each panel indicate raw data points. Lower case letters indicate significant (*p* < 0.05) differences among sites as determined using post-hoc tests. Data are presented as mean ± SEM.

Among seawater samples, the concentrations of both total *Vibrio* (ANOVA, F_2,6_ = 8.3, *p* < 0.05) and *V. harveyi* (ANOVA, F_2,6_ = 206, *p* < 0.001) differed significantly between sites. There was a significant and positive correlation between recorded oyster mortality and both total *Vibrio* (R^2^ = 0.51, *p* < 0.05) and *V. harveyi* (R^2^ = 0.47, *p* < 0.05) abundances. Total *Vibrio* abundance was significantly higher at Karuah River and Tilligerry Creek relative to Cromarty Bay (Tukey HSD, *p* > 0.05). Concentrations of *V. harveyi* were greatest at the high mortality site, Tilligerry Creek. Cromarty Bay (low mortality) had significantly lower concentrations of *V. harveyi* compared to both other sites (Figure 7).

*V. harveyi* abundance in oyster biofilms differed significantly between sites (ANOVA, F_2,9_ = 4.3, *p* < 0.05), mirroring patterns in oyster mortality. There were higher abundances of *V. harveyi* in Tilligerry Creek (high mortality) relative to Cromarty Bay (low mortality) (Tukey HSD, *p* < 0.05). Total *Vibrio* abundances within oyster biofilms also differed significantly between sites (ANOVA, F_2,15_ = 5.4, *p* < 0.05) with *Vibrio* abundances higher in oysters collected from Tilligerry Creek relative to Karuah River (Tukey HSD, *p* < 0.05; Figure 7). *V. harveyi* and total *Vibrio* concentrations in oyster gill tissue samples did not differ among sites (ANOVA, *p* > 0.05).

*V. parahaemolyticus* was only detected in seawater samples, and not detected in tissue or biofilm samples. *V. parahaemolyticus* in seawater was significantly greater in abundance at Cromarty Bay (Mean ± SE = 7185 ± 1008) compared to Karuah (Mean ± SE = 1471 ± 371) (ANOVA, F_2,3_ = 23.5, *p* < 0.05).

### Subsequent sampling

**Figure 8:**
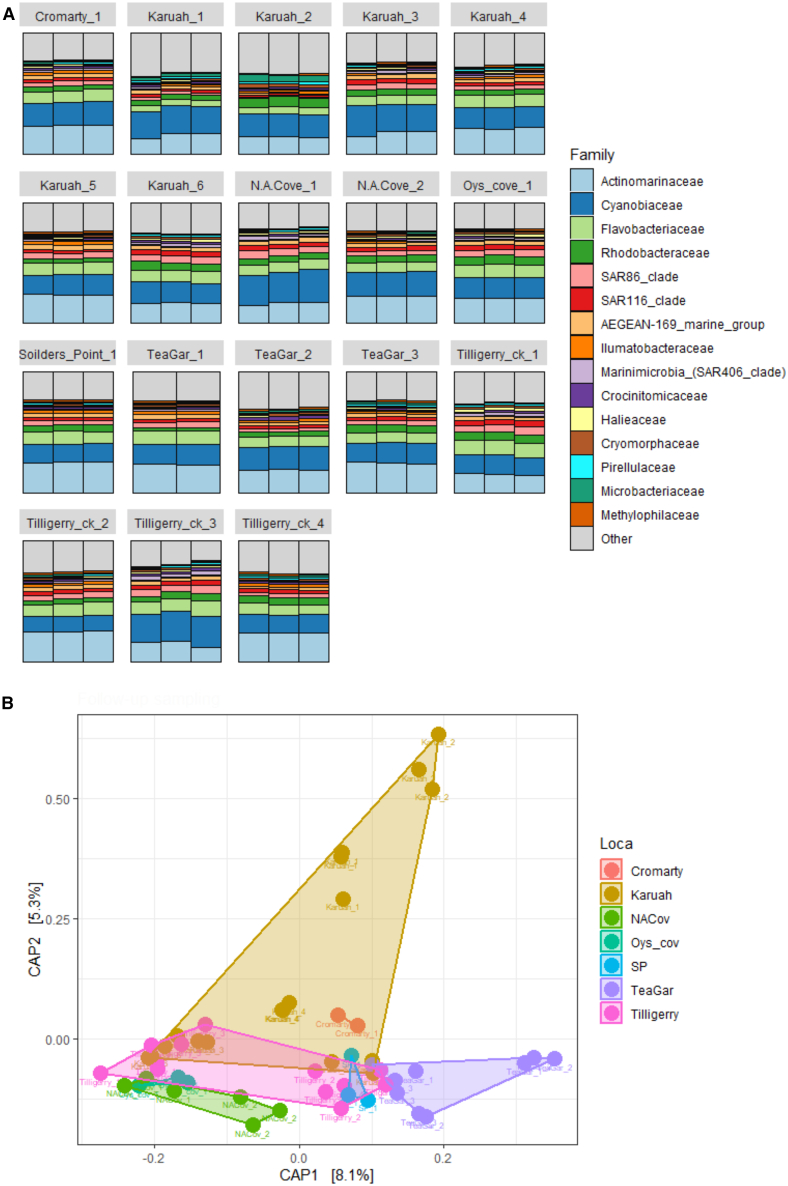
Bacterial communities from subsequent sampling (A) Barplot of the top 20 most abundant bacterial families in Seawater from Intensive subsequent sampling. Each bar represents an individual sample, bars are grouped by locations. (B) CAP ordination plots calculated from Weighted Unifrac distances of Seawater bacterial communities. Locations are indicated by colors.

Intensive spatial sampling of the seawater in the weeks following oyster mortality revealed significant differences among locations (PERMANOVA, F_6,52_ = 1.9, *p* < 0.05) and sites in the microbial community composition (PERMANOVA, F_17,52_ = 4.2, *p* < 0.05; Figure 8). Furthermore, there were significantly different microbial communities in seawater samples at this time compared to the on-farm sampling conducted during the period of high oyster mortality (February) (PERMANOVA Location × Sampling time; F_2,41_ = 2.6, *p* < 0.05). When comparing the two sampling events, we found that Karuah and Cromarty Bay had not significantly changed in their microbial community composition, however, the bacterial community composition of Tilligerry creek (high mortality) was significantly different between sampling times (PERMANOVA; F_1,14_ = 3.1, *p* < 0.05). We found 12 ASVs were in greater abundance at the time of follow-up sampling, 6 of which were from the *Synechococcales* order (DESEQ, P_adj_ < 0.05). There was also 12 ASVs there were more abundant at the time of on-farm sampling, six of which were from the *Flavobacteriales* order (DESEQ, P_adj_ < 0.05).

**Figure 9:**
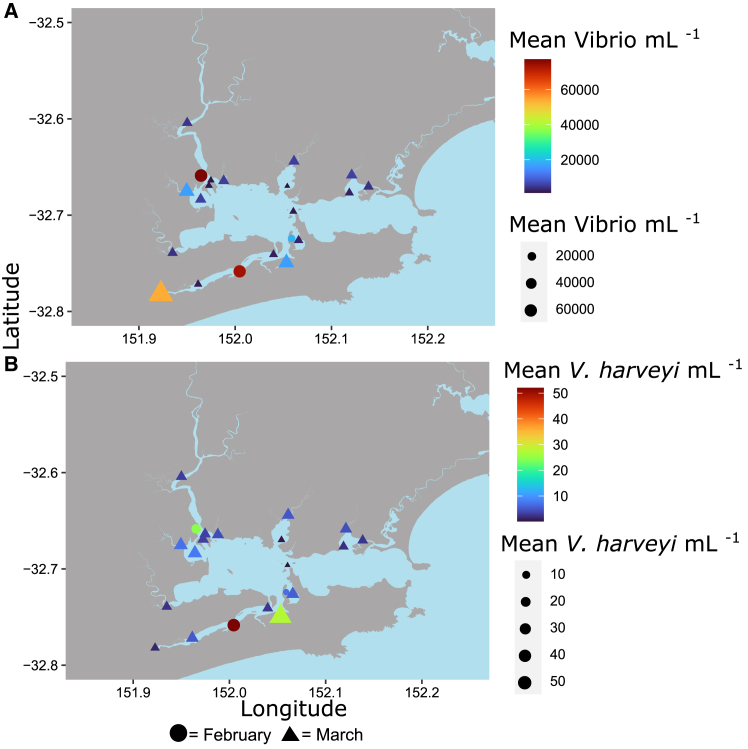
Bacterial abundances from subsequent sampling Total *Vibrio* abundance mL^−1^ (A) and *V. harveyi* abundance mL^−1^. (B) From seawater samples collectes in Febuary (on-farm sampling, circles) and March (Intensive subsequent sampling, triangles). Both the size and color of shapes indicates the abundance of bacteria at each sampling site. Data are presented as mean ± SEM.

**Figure 10:**
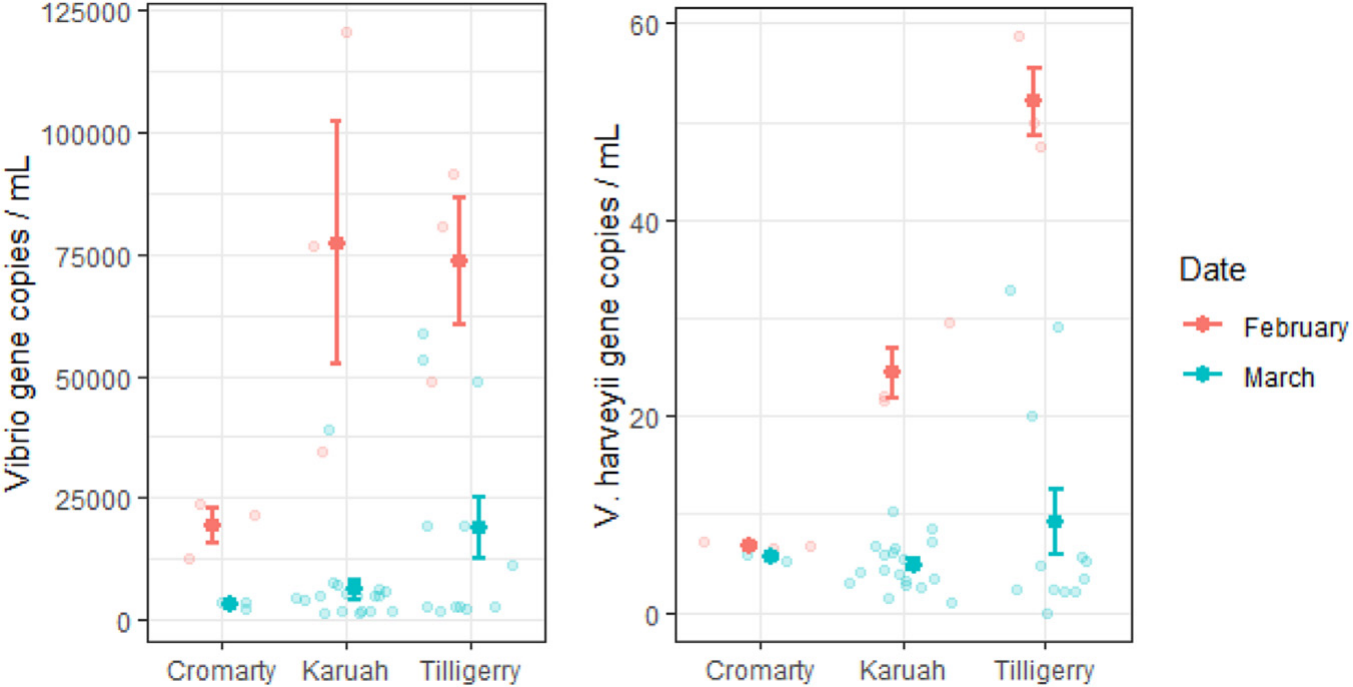
*Vibrio* (left) and *V. harveyi* (right) mL^−1^ in each of the three locations that were sampled in both February (orange) and March (green) 2023 Data are presented as mean ± SEM. Small points indicate raw data.

Total *Vibrio* (ANOVA, F_17,36_ = 21.6, *p* < 0.001) and *V. harveyi* (ANOVA, F_17,36_ = 2.8, *p* < 0.01) abundances differed significantly across the 16 sites. Notably, the highest levels of both total *Vibrio* (Tukey HSD) and *V. harveyi* occurred within the high oyster mortality site of Tilligerry Creek. Furthermore, both total *Vibrio* and *V. harveyi* abundances were significantly greater during the February (high mortality) than March (low mortality) sampling periods (ANOVA Date x Site; F_2,36_ = 4, *p* < 0.05; ANOVA Date x Site; F_2,36_ = 18.4, *p* < 0.001) at the two sites where high levels of oyster mortality were recorded (Tilligerry Creek and Karuah River) (Figure 9). Indeed, *V. harveyi* abundance at these sites was approximately five times greater during the mortality event. However, at Cromarty Bay, where there was almost no oyster mortality recorded, there were no significant differences in either total *Vibrio* or *V. harveyi* abundance in seawater samples between February and March (Figure 10). *V. parahaemolyticus* did not significantly differ among the sites (ANOVA *p* > 0.05), however, there was significantly (ANOVA F_1,33_ = 4.7, *p* < 0.05) greater abundances of *V. parahaemolyticus* in March (Mean ± SE = 14,215 ± 3687 copies mL^−1^) compared to February (Mean ± SE = 3350 ± 1251 copies mL^−1^). This pattern in *V. parahaemolyticus* abundance is the opposite to those observed for both total *Vibrio* and *V. harveyi* abundances.

When investigating the relationships between *Vibrio* abundance and environmental variables, the top three predictors of total *Vibrio* abundance were Chlorophyll *a* (10.5% inc. MSE), Dissolved Oxygen (10.5% inc. MSE) and Salinity (9.5% inc. MSE). Chlorophyll *a* showed a positive relationship with *Vibrio* abundance, while both Oxygen (10.5% inc. MSE) and Salinity (9.5% inc. MSE) had a negative relationship. RF modeling revealed the top predictors of *V. harveyi* abundance were Chlorophyll *a* (12.4% inc. MSE), Turbidity (11.7% inc. MSE), Dissolved Oxygen (10.8% inc. MSE) and Temperature (10.2% inc. MSE). The top predictor for *V. parahaemolyticus* abundance was Temperature (11.3% inc. MSE) which showed a positive relationship with *V. parahaemolyticus* abundance. This was followed by Chlorophyll *a* (9.9% inc. MSE) and turbidity (9.8% inc. MSE) which were both also positively correlated with *V. parahaemolyticus* abundance.

## Discussion

Large-scale oyster mortality events and farm closure due to food-poisoning have detrimental impacts on the global oyster aquaculture industry,^4^ with bacteria often identified as putative causative agents.^21^^,^^36^ We monitored putative bacterial pathogens of both oysters and humans from the *Vibrio* genus over a period of elevated oyster mortality in the summer of 2022–2023 in Australia. Commercial losses from this mortality event were estimated to be in the range of $5–10 million AUD (personal communication with oyster farmers). Here, we present a comprehensive analysis of potential bacterial pathogens in seawater before, during and after an extensive oyster mortality in one of the Australia’s most productive oyster aquaculture regions.

### *V. harveyi* in the seawater timeseries

*V*. *harveyi* is a known pathogen of aquatic organisms including oysters.^37^ This pathogen was also identified in elevated abundance in oyster tissues from Port Stephens at sites experiencing mortality in 2013.^7^ Because of *V. harveyi’s* previous implication in Port Stephens oyster mortality, we focused on quantifying planktonic *V. harveyi.* Planktonic populations of *V. harveyi* displayed clear cyclical patterns of abundance that reflected annual SST trends. The optimum temperature for *V. harveyi* has been reported to be 26°C^18^, and previous studies have reported increases in *V. harveyi* abundances and virulence when SST is elevated.^18^^,^^38^ However, to our knowledge, our study is the first to identify such clear seasonal links between water temperature and *V. harveyi* abundance over multiple years.

There is an established link between *V. harveyi* abundance in oyster tissue, warming, and oyster mortality.^7^^,^^12^^,^^17^ Our study, however, provides new evidence for a significant link between the planktonic abundances of *V. harveyi* and oyster mortality. Outbreaks of *C. gigas* mortality often coincide with warmer SSTs, with mass mortality events during warmer periods often loosely termed “summer mortality”, which have been recorded globally,^8^^,^^10^^,^^39^ including in this region of Australia.^7^^,^^12^ While summer mortality events have been linked to multiple causative agents,^39^ recent research has highlighted a potential role of *V. harveyi*.^12^^,^^17^ We saw significant increases in total *Vibrio* as well as *V. harveyi* during oyster mortality, indicating that there may be other *Vibrio* pathogens affecting oysters in addition to *V. harveyi*. Our results demonstrate planktonic *V. harveyi* may also be implicated in Pacific oyster mortality.

### Elevated *V. harveyi* during oyster mortality

During a period of severe Pacific oyster mortality in Port Stephens, levels of *V. harveyi* were elevated in both the planktonic phase and associated with oyster biofilms. At Tilligerry Creek, the epicenter of the oyster mortality event, there was significantly elevated *Vibrio* and *V. harveyi* abundances, in addition to a significantly altered bacterial community composition. Subsequent sampling after the peak of the mortality event had passed revealed a significant decrease in *V. harveyi* abundance at the locations where mortality was greatest (Tilligerry Creek and Karuah River), while there was no change in abundance at the site with almost no oyster mortality (Cromarty Bay). Together these results point toward a link between oyster mortality and the presence of *V. harveyi*.

Our findings add to a growing body of evidence that implicate *V*. *harveyi* in oyster mortality events, both in Australia^7^^,^^15^^,^^17^ and around the globe.^3^^,^^8^ *V. harveyi* has been implicated in *C. gigas* mortality during summer in China, and was highlighted as a bacterial species that should be surveyed in an attempt to reduce the risk of summer mortality.^8^ A previous investigation of *C. gigas* summer mortality in Port Stephens also highlighted increases in *Vibrio*, including *V. harveyi* within *C. gigas* associated bacterial communities at sites affected by oyster mortality.^7^^,^^12^ *V*. *harveyi* can also trigger mortality in other mollusc species. For example, the abalone *Halotis tuberculata* in France was found to be highly susceptible to mortality caused by *V*. *harveyi* during summer when seawater temperatures were greatest.^40^

Recent research has also linked *C. gigas* mortality to a marine heatwave. Research performed in Sydney, 200 km south of Port Stephens, found that during a marine heatwave, elevated *C. gigas* mortality co-occurred with increased abundances of *V. harveyi* and other *Vibrio* species.^12^ This is consistent with evidence from two simulated heatwave studies. The first study investigated the effects of a marine heatwave on *C. gigas*,^15^ and the second study investigated the effects of an atmospheric heatwave on the Sydney rock oyster *Saccostrea glomerata*.^17^ Both studies found that a heatwave (either marine or atmospheric) increased oyster mortality, and concurrently, the abundance of *V. harveyi* in oysters. The observation that mortality could be prevented in these studies by administering antibiotics further highlighted the likely role of these bacteria in oyster mortality.^15^^,^^17^

In this study, we observed orders of magnitude elevation in planktonic abundances of both total *Vibrio* and *V. harveyi* abundances in seawater during a period of oyster mortality. Our observations are notable because while there is evidence for increases in the abundance of *Vibrios* within oyster tissue during oyster mortality events,^2^^,^^3^^,^^7^^,^^15^ few studies have linked the planktonic abundance of *Vibrio* and more specifically, *V. harveyi,* to oyster mortality. These findings reveal the potential importance of shifts in planktonic bacterial reservoirs within oyster mortality events.

Intriguingly, we found that while abundances of total *Vibrio* and *V. harveyi* were elevated in both the seawater and external biofilms during periods, and in locations, of high oyster mortality, they were not significantly elevated in oyster gill tissue. This, however, may be an example of survivor bias,^41^ whereby oysters were sampled in the midst of a mortality event where >70% of the *C. gigas* on farms had died and the remaining pool of oysters that were sampled may have been resistant or had already overcome *Vibrio* infection. There is a known genetic basis to defense from bacterial infection and there is genetic variability among the oyster populations,^42^^,^^43^^,^^44^ meaning that these remaining survivors may have been capable of preventing the proliferation of *Vibrio* within their tissues.^15^^,^^17^

### V. parahaemolyticus

*V*. *parahaemolyticus* is a pathogen affecting human consumers of oysters.^45^^,^^46^ We investigated its presence in this study because the extensive sampling provided an ideal opportunity to investigate *V. parahaemolyticus* in the rapidly warming estuaries on Australia’s eastern coastline.^42^ While *V. parahaemolyticus* is not considered a pathogen of oysters, it can still cause economic damage to in the industry due to immediate closure of oyster sales and ongoing damage to the product image.^47^ For example, an outbreak of 250 cases of *V. parahaemolyticus* infection in Australia in 2021^48^ shutdown the Pacific oyster industry in a major oyster harvesting region for four months, affecting at least 30 businesses and costing one business $1.7 million AUD; however, the total economic cost is not known.^49^

Rising global seawater temperatures have been flagged as potentially increasing the abundance of marine pathogens affecting humans, including *V. parahaemolyticus*.^24^ In addition to these rising temperatures, marine heatwaves are increasing in frequency and severity^50^ with unknown consequences for marine diseases. One of the first serious detections of *V. parahaemolyticus* affecting the Sydney rock oyster *Saccostrea glomerata* coincided with a marine heatwave off the south eastern Australian coast in 2024.^51^ Indeed, we found *V. parahaemolyticus* to be most abundant in March when water temperatures are greatest, and also found temperature to be the strongest predictor of its abundance. Our time series data showed that *V. parahaemolyticus* followed a cyclical pattern of abundance, similar to *V. harveyi* whereby greatest abundances occurred toward the end of summer.

### Environmental indicators

The environmental factors that influenced planktonic abundances of *Vibrio*, *V. harveyi,* and *V. parahaemolyticus* in this region included phytoplankton abundance, as indicated by both satellite-derived Chlorophyll *a* levels and chloroplast counts in 16S rRNA amplicon sequencing analysis, temperature, salinity, and turbidity. A range of both biotic and abiotic factors have previously been described to influence planktonic *Vibrio* abundance. Drivers such as temperature and salinity are well established to control *Vibrio* growth,^52^^,^^53^ but also to govern the growth of other members in their planktonic community. Plankton provides a nutrient rich habitat favoring heterotrophic bacteria, including *Vibrios*.^54^^,^^55^ The potential interaction between phytoplankton and *Vibrio* abundance warrants further investigation because oyster farmers often place oysters in areas with high phytoplankton productivity to enhance oyster growth rates.^56^

We identified an annual cyclical dynamic in planktonic *Vibrio* abundance and especially strong patterns of abundance for *V. harveyi* and *V. parahaemolyticus* where the abundance of these bacteria peaked during the late summer-early autumn months (February - April) when SSTs in the Port Stephens region were greatest. Elevated planktonic *Vibrio* concentrations have previously been found to coincide with elevated SSTs,^57^^,^^58^ but to our knowledge, our study is the first to demonstrate this seasonal pattern in the putative pathogen *V. harveyi* and the human pathogen *V. parahaemolyticus* over multiple years. We suggest that Elevated SST and chlorophyll *a* may provide oyster farmers with an indication that potentially pathogenic *Vibrio* are also likely to be elevated and oyster mortality could occur.

### Conclusions

Here we combined analysis of temporal patterns in planktonic bacteria, including putative pathogens from the *Vibrio* genera, with an analysis of environmental conditions during an oyster mortality event within one of the Australia’s most productive oyster cultivation regions. We observed a clear seasonal pattern in *Vibrio*, *V. harveyi,* and *V. parahaemolyticus* abundance, with abundances peaking during late summer. We also observed significant spatial and temporal correlations between the abundance of *V. harveyi* and oyster mortality. While these correlations cannot prove causation, the patterns observed here, when combined with other evidence in the literature^2^^,^^7^^,^^12^^,^^15^^,^^17^^,^^59^ points toward a potential role of planktonic *V. harveyi* in a significant Pacific oyster mortality event. We contend that environmental triggers for increases in *V. harveyi* and *V. parahaemolyticus* abundance, including warming waters and increased phytoplankton biomass, might provide proxy measures for risk that oyster farmers could apply to guide the management of oyster stocks.

### Limitations of the study

This study was conducted in the natural environment and did not manipulate temperature or other variables. This could be viewed as a limitation because our findings could be co-correlated with other variables that were not measured.

## Resource availability

### Lead contact

Further information and requests for resources and reagents should be directed to and will be fulfilled by the lead contact, Elliot Scanes (elliot.scanes@uts.edu.au).

### Materials availability

This study did not generate new unique reagents.

### Data and code availability

• All 16s gene sequences have been deposited at the NCBI data repository and are publicly available as of the date of publication. Accession numbers are listed in the key resources table. All remaining data can be shared by the lead contact following request.
• This study did not generate any original code.
• Any additional information required to reanalyze the data reported in this article is available from the lead contact upon request.

## Acknowledgments

This project was funded by 10.13039/501100000923Australian Research Council grants awarded to J.R. Seymour & M. Labbate (grant ID: DP210101610) and E. Scanes & J.R. Seymour (grant ID: DP240100370). We would like to acknowledge the assistance of Matt Burgoyne and Garth Richards for their assistance collecting samples.

## Author contributions

Conceptualisation, E.S., N.S., M.L., and J.R.S.; methodology, E.S., N.S., M.L., and J.R.S.; investigation, E.S., N.S., J.P., and S.R., resources, E.S., N.S., and J.R.S.; writing – original draft, E.S.; writing – review and editing, E.S., N.S., J.P., S.R., M.L., and J.R.S.; visualization, E.S. and S.R.

## Declaration of interests

Elliot Scanes reports financial support was provided by Australian Research Council. Justin Seymour reports financial support was provided by Australian Research Council. Maurizio Labbate reports financial support was provided by Australian Research Council. The other authors declare that they have no known competing financial interests or personal relationships that could have appeared to influence the work reported in this article.

## STAR★methods

### Key resources table

**Table:** 

REAGENT or RESOURCE	SOURCE	IDENTIFIER
**Deposited data**		

16S sequence data	NCBI Sequence Read Archive (SRA)	PRJNA1173889

### Experimental model and study participant details

This study collected Pacific oysters *Crassostrea gigas* from the stock of commercial oyster farms in Port Stephens, New South Wales, Australia. The care and husbandry of oysters was undertaken as part of normal oyster farming operations and was therefore not part of this study.

### Method details

This study was performed in Port Stephens, a warm-temperate/sub-tropical estuary in eastern Australia that on average produces approximately 12 million oysters each year.^34^ Despite previously high levels of Pacific oyster mortality in this environment,^7^ monitoring performed outside of this study by NSW government agencies has found that OSHV-1 is not present in oysters from Port Stephens, and potential pollutants including pesticides and heavy metals are not elevated beyond normal ranges.^60^ The three-pronged sampling regime used here incorporated (i) a 27-month timeseries of seawater sampling; (ii) reactive sampling during a major Pacific oyster mortality event; and (iii) spatially intensive sampling in the period immediately following the oyster mortality event.

#### Seawater time series

Triplicate 2 L seawater samples were collected from the upper 0.5 m of the water column weekly from the seawater environment adjacent to an oyster hatchery in Port Stephens (−32.714393 °S, 152.180204 °E) from 27/2/2020 to 7/5/2022. Samples were immediately filtered onto 0.22 μm membrane filters (Millipore, DURAPORE PVDF 0.22μm, 47 mm) using a peristaltic pump. Filters were frozen at −20°C until DNA extraction.

Throughout the time-series, sea surface temperature (SST) and Chlorophyll-a concentration was obtained from the Australian Ocean Data Network portal (AODN). SST data was obtained from satellite data, using the IMOS SRS L3S night time SST data for the closest grid cell (1.1 × 1.1 km) to sample collection, whereby each grid cell contains the one-night average of all the highest available quality SSTs that overlap with that cell. Mean temperature for the week during which samples were collected was then calculated. Chlorophyll *a* (mg/m^3^) concentrations were also obtained from satellite data using the IMOS SRS MODIS multispectral sensor from the same grid cell (1.1 × 1.1 km) as SST. Chlorophyll *a* data was determined using the OC3 method recommended by the NASA Ocean Biology Processing Group and implemented in the SeaDAS processing software l2gen. The OC3 algorithm is described at http://oceancolor.gsfc.nasa.gov/cms/atbd/chlor_a (IMOS 2023).

#### On-farm oyster sampling

In February 2023, oyster farmers in Port Stephens reported significant Pacific oyster mortality at some locations. One week after oyster mortality was first observed, mortality was self-reported by farmers and recorded as the ratio of alive vs. dead in oyster cultivation baskets in three locations; Tilligerry Creek (−32.756439° S, 152.008178° E), Karuah River (−32.656932° S, 151.968206° E), and Cromarty Bay (−32.723083° S, 152.06076° E). Farmers also observed the development of a distinct biofilm present on oyster shells at the sites where elevated levels of mortality were recorded.

To characterise bacterial communities and abundance at oyster farms during the mortality event, seawater, oyster shell biofilm and oyster gill tissue samples were collected. From each of the three locations (Figure 1), triplicate 2 L water samples were collected from the upper 0.5 m of the water column and filtered on site onto 0.22 μm membrane filters (Millipore, DURAPORE PVDF 0.22 μm, 47 mm) using a peristaltic pump. Filters were placed into a cryovial and snap frozen in liquid nitrogen and stored at −80°C until DNA extraction. At each location (Figure 1), six live oysters were also collected from oyster cultivation baskets and then gently rinsed in seawater from the site to remove any loose sediment adhered to the external shell. Oysters were then immediately placed into individual zip-seal plastic bags. Once back on shore, biofilm and gill tissue samples were taken. To collect biofilm samples, each oyster was swabbed using two sterile disposable inoculation loops along the length of the shell ten times (ten times per loop), before the loops were cut 20 mm from the end and placed into a cryovial (*n* = 6). Cryovials were then snap frozen in liquid nitrogen and later stored at −80°C until DNA extraction. For gill tissue samples, each oyster was shucked and the gills were extracted using sterile tweezers and scissors (*n* = 6). Gill tissue was placed into a cryovial, snap frozen in liquid nitrogen and stored at −80°C until DNA extraction. Gill tissue was selected because it has previously provided a good indicator of microbial shifts correlating with oyster mortality.^17^ Each individual oyster was treated as a biological replicate at each site.

#### Intensive subsequent sampling

One month after the on-farm oysters sampling, spatially intensive sampling was conducted over two days in March 2023 (2/3/2023-3/3/2023). Eighteen sites were selected throughout the Port Stephens estuary, encompassing the locations that experienced significant oyster mortality (Tilligerry Creek and Karuah), in addition to sites that experienced low mortality (Cromarty Bay) and additional control sites (Figure 1). At each of the 18 sites, triplicate 2 L seawater samples were collected from the upper 0.5 m of the water column and filtered onto 0.22 μm membrane filters (Millipore, DURAPORE PVDF 0.22 μm, 47 mm) using a peristaltic pump. Filters were then immediately frozen at −20°C until DNA extraction. At each site a vertical profile of temperature (°C), salinity (ppt), conductivity (μS/cm), pH, dissolved oxygen (% sat), and turbidity (NTU) was measured using a YSIEXO 2 multiparameter water quality sonde (Table S1).

#### DNA extraction

DNA was extracted from filter membranes using the Qiagen DNeasy PowerWater Kit following all manufacturer’s instructions. Tissue and external shell biofilm samples were extracted using the Qiagen DNeasy Blood & Tissue Kit following all manufacturer’s instructions. Three blank samples from each DNA extraction kit were extracted alongside the samples. All extracted DNA was stored at −30°C for downstream analysis.

#### 16S rRNA amplicon sequencing

To characterise bacterial community composition from all samples, the V3–V4 region of the bacterial 16S rRNA gene was amplified using the Bakt_341F and Bakt_805R primer set,^61^ with the following cycling conditions: 95°C for 3 min followed by 25 cycles of: 95°C for 30 s, 55°C for 30 s, 72°C for 30 s, and then 72°C for 5 min with a final hold at 4°C. Amplicon sequencing of the 16S rRNA was performed using the Illumina NovaSeq SP Lane, 500 cycle following the manufacturer’s guidelines (Australian Genome Research Facility (AGRF), Melbourne, Australia). Raw data files in FASTQ format were deposited in NCBI Sequence Read Archive (SRA) under Bioproject number PRJNA1173889.

Raw demultiplexed 16S rRNA data was processed using the Quantitative Insights into Microbial Ecology (QIIME 2 version 2019.1.0) pipeline. Briefly, paired-end sequences were imported, trimmed and denoised using DADA2 (version 2020.6).^62^ Bacterial sequences were identified at the single nucleotide level (Amplicon Sequence Variants; ASV), chloroplasts were removed and taxonomy was assigned using the classify-sklearn qiime feature classifier against the Silva v138 database.^63^ Rarefaction plots were used to check sequencing depth, and data from the seawater timeseries were rarefied to 13100 cleaned and joined reads per sample, and data from the on-farm and subsequent sampling were rarefied to 7300 read per sample.

#### Quantitative PCR

Abundances of total *Vibrio* were quantified using quantitative PCR (qPCR) with the primer pair Vib1-f and Vib2-r,^64^^,^^65^ which targets the 16S rRNA gene specific to the *Vibrio* genus. To quantify the abundances of *V. harveyi* we used the primer pair of mreB11F and mreB9bisR,^66^ which targets the *mreB* protein that has previously been aligned to 171 *V harveyi* strains from NCBI. We then constructed a TaqMan probe 5′-FAM-AACTACGGCAGCTTGATCGGTGAA-ZEN-IBFQ-3′ on a conserved area between the two primers. For direct quantification of *V. parahaemolyticus* we targeted the *tlh* gene using the TLH-F (5′-AAAGCGGATTATGCAGAAGCACTG-3′) and TLH-R (5′-TGTGCCTTGATGAACTCGTTC-3′) primers^67^^,^^68^ together with the TLH-P (5′- TCGTTTGACGGACGCAGGTGCGAAGAA-3′) probe.^69^

Quantitative PCR (qPCR) assays were prepared with an epMotion 5075I Automated Liquid Handling System and performed on a Bio-Rad CFX384 Touch Real-Time PCR Detection System, with three technical replicates, a standard curve, and negative controls. A melting curve was also added to the end of the *Vibrio* specific SYBR assay to confirm the presence of a single PCR product. The reaction mixture for each assay included: 2.5 μL iTaq Universal probes SMX or iTaq Universal SYBR Green SMX (Bio-Rad), 0.2 μL of each 10 μM forward and 10 μM reverse primer, 0.1 μL of 10 μM probe (for the *mreB* and *tlh* assays), 1 μL of template DNA, and 1 or 1.1 μL of sterile water for a final reaction volume of 5 μL. For 16S and *V. harveyi* assays, qPCR was performed using the following cycling conditions: 95°C for 3 min followed by up to 45 cycles of 95°C for 15 s and 60°C for 1 min. The *V. parahaemolyticus* assay was identical, but with an annealing extension step at 62°C for 60 s.

Each oyster sample was run in three technical replicates. For quality control, the coefficient of variation percentage (%CV) was calculated for the qPCR Quantification Cycle (Cq) technical triplicates. If the %CV exceeded 2%, one technical replicate was removed from the analysis. Additionally, any biological samples with a Cq %CV greater than 5% were excluded from the analysis. The resulting data were normalised to gene copies per volume of water.

### Quantification and statistical analysis

Bacterial community composition data from the seawater time series was analyzed using PERMANOVA on weighted unifrac distance matrices. PERMANOVA analyses were performed with both Temperature, Chlorophyll-a or Chloroplast as factors. DESEQ analysis was used to determine which taxa were significantly affected. Random forest (RF) regression analysis was used to predict which factors were most important in determining total *Vibrio V. harveyi* and *V. parahaemolyticus* abundance from seawater time series samples. Predictor variables in RF analysis were SST, Chloroplast, Date, and Chlorophyll *a*. To model cyclical patterns of bacterial abundances in our seawater time series, a Generalised Additive Model (GAM) was used with “Day” as a smoothing term, and “Year” as a categorical term.

Bacterial communities from on-farm samples and intensive subsequent samples were analyzed for differences among Locations using PERMANOVA calculated from Weighted unifrac distances and with “Location” as a single fixed factor. Separate analyses were done for each on-farm sample type (Biofilm, Gill and Seawater). A separate PERMANOVA calculated from weighted unifrac distances was performed to compare sites with “Site” as the single fixed factor for intensive subsequent samples. A non-parametric Kruskal Wallace test was used to determine differences in the abundance of the top 15 most abundant *Vibrio* ASVs from our sequencing data. Dunn’s test was used post-hoc to determine pairwise differences.

*Vibrio* and *V. harveyi* abundance from Port Stephens in seawater, tissue and shell biofilm samples from on-farm sampling was analyzed using ANOVA after it was confirmed that the data was normally distributed using Shapiro’s test. Differences among sites were determined using a single factor ANOVA with “Site” as the factor. Significant differences among sites were tested using Tukey post-hoc tests.

RF models were used to predict *Vibrio*, *V. harveyi* and *V. parahaemolyticus* abundance from Intensive subsequent sampling with the predictors; temperature (°C), Chlorophyll *a* (μg L^−1^), dissolved Oxygen (mg L^−1^), turbidity (NTU) and salinity (PSU).

Intensive subsequent sampling data was compared to on-farm data by selecting the subset of sites that were within the same bay or tributary of Port Stephens as the on-farm samples (Figure 1). The bacterial communities of these subsets were then compared using PERMANOVA, calculated from Weighted unifrac distances and with “Location” as the first factor (Cromarty, Karuah, Tilligerry) and “Sampling time” (On-Farm and Intensive subsequent) as the second factor.

Abundances of *Vibrio* and *V. harveyi* were compared in seawater samples between on-Farm and Intensive subsequent samples using a two-factor ANOVA with the factors “Site” (Tilligerry Creek, Karuah River and Cromarty Bay) and Sampling “time” (On-Farm and Intensive subsequent). Post-hoc Tukey Tests were used to determine the source of variation among Sites. All data analysis was performed using R software.

## Supplemental information

Table S1. Table describing the attributes and locations of sites from this study
