# Supplementary material for: Temporal and spatial co-occurrence of pacific oyster mortality and increased planktonic Vibrio abundance
Source: iScience. 2024 Dec 21;28(2):111674. doi: 10.1016/j.isci.2024.111674 (PMC11787541; doi:10.1016/j.isci.2024.111674)
Supplement: Table S1. Table describing the attributes and locations of sites from this study [file mmc1.pdf]

iScience, Volume *28*

Supplemental information

Temporal and spatial co-occurrence of pacific oyster mortality and increased planktonic *Vibrio* abundance

Elliot Scanes, Nachshon Siboni, Jaimie Potts, Shivanesh Rao, Maurizio Labbate, and Justin R. Seymour

Full name	Tilligerry_ck_1<br>Tilligerry creek 1	Tilligerry_ck_2<br>Tilligerry creek 2	Karuah_1<br>Karuah River 1	Karuah_2<br>Karuah River 2	Karuah_3<br>Karuah River 3	Karuah_4<br>Karuah River 4	Karuah_5<br>Karuah River 5	Karuah_6<br>Karuah River 6	Oys_cove_1<br>Oyster Cove 2
Temp °C	25.72	25.76	27.12	26.48	26.39	27.74	26.46	27.49	25.91
DO % sat	90.29	89.29	88.79	88.43	87.37	93.26	84.29	94.60	85.12
DO mg/L	6.11	5.97	6.01	5.89	5.84	6.02	5.56	6.14	5.65
SpCond µS	50207.79	53234.35	44555.01	50949.09	50728.45	53591.01	53565.70	53431.80	54449.73
EC µS	50901.61	54009.93	46359.90	52386.03	52074.45	56396.28	55056.34	55973.43	55396.59
Salinity psu	32.87	35.11	28.73	33.40	33.24	35.32	35.34	35.21	36.01
pH	7.99	8.08	7.81	7.99	7.93	8.02	7.98	7.97	7.93
Turbidity NTU	1.45	0.92	1.25	1.20	1.21	9.34	6.44	5.39	3.68
Chl-a µg/L	5.11	2.20	4.18	2.11	1.81	4.98	5.54	3.09	3.67
fDOM RFU	20.28	7.91	14.33	8.22	8.66	10.40	10.59	8.39	11.38
fDOM QSU	61.93	24.13	43.75	25.06	26.42	31.74	32.30	25.60	34.73
Lattitude	-32.7717575	-32.7408146	-32.6041832	-32.6693253	-32.6638102	-32.6752091	-32.6835843	-32.6644117	-32.7392375
Longitude	151.9614519	152.0398043	151.9501295	151.9727215	151.9747463	151.9495898	151.9641583	151.9880778	151.9349299

Full name	Tilligerry_ck_3<br>Tilligerry Creek 3	N.A.Cove_1<br>North Arm Cove 1	N.A.Cove_2<br>North Arm Cove 2	TeaGar_1<br>Tea Gardens 1	TeaGar_2<br>Tea Gardens 2	TeaGar_3<br>Tea Gardens 3	Tilligerry_ck_4<br>Tilligerry Creek 5	Soilders_Point_1<br>Soilders Point 1	Cromarty_1<br>Cromarty Bay 1
Temp °C	25.02	28.71	26.10	25.44	25.00	24.67	25.19	24.73	25.50
DO % sat	52.25	106.43	99.74	78.07	93.60	95.11	85.14	97.93	90.12
DO mg/L	3.68	6.74	6.62	5.23	6.33	6.46	5.74	6.65	6.04
SpCond µS	43545.65	54506.35	53576.22	53832.47	53562.95	53496.79	53400.18	53400.41	53726.66
EC µS	43564.79	58371.17	54698.26	54284.57	53558.19	53164.52	53589.84	53123.49	54242.94
Salinity psu	28.05	35.97	35.35	35.56	35.37	35.33	35.24	35.26	35.48
pH	7.45	8.07	8.22	8.04	8.19	8.23	8.08	8.21	8.10
Turbidity NTU	3.03	5.33	1.18	1.83	1.35	0.75	2.16	0.50	2.76
Chl-a µg/L	11.59	5.68	2.02	4.08	2.81	1.68	2.43	2.07	6.64
fDOM RFU	35.63	7.38	2.72	5.14	2.74	1.92	5.92	2.11	4.07
fDOM QSU	108.83	22.52	8.28	15.66	8.33	5.82	18.05	6.40	12.41
Lattitude	-32.781983	-32.6440942	-32.6698265	-32.6584823	-32.6703463	-32.6768032	-32.7493918	-32.6961735	-32.7261167
Longitude	151.9228022	152.0610541	152.0540934	152.1210256	152.1384459	152.1185606	152.0531792	152.0604059	152.0658494
